# Dental implants and grafting success remain high despite large variations in maxillary sinus mucosal thickening

**DOI:** 10.1186/s40729-017-0064-8

**Published:** 2017-01-18

**Authors:** Bartosz Maska, Guo-Hao Lin, Abdullah Othman, Shabnam Behdin, Suncica Travan, Erika Benavides, Yvonne Kapila

**Affiliations:** 1 Department of Periodontics and Oral Medicine, School of Dentistry, University of Michigan, 1011 N University Ave, Ann Arbor, MI USA; 2 Department of Surgical Sciences, School of Dentistry, Marquette University, 1801 W Wisconsin Ave, Milwaukee, WI USA; 3 Department of Periodontology & Dental Hygiene, University of Detroit Mercy, 2700 Martin Luther King Jr. Blvd, Detroit, MI USA; 4 Department of Periodontics, School of Dental Medicine, Case Western Reserve University, 2124 Cornell Rd, Cleveland, OH USA; 5 Department of Orofacial Sciences, School of Dentistry, University of California San Francisco, 513 Parnassus Ave, S612D, Box 0422, San Francisco, 94143 CA USA

**Keywords:** Mucosal thickening, Dental implants, Maxillary sinus, Sinus floor augmentation, Periodontal diseases, Cone-beam computed tomography

## Abstract

**Background:**

Although mucosal thickening is the most common radiographic finding observed regarding sinus pathology, the knowledge regarding its clinical significance on the outcomes of dental implants and grafting in the maxillary sinuses is still limited. We hypothesized that mucosal thickening would not alter the predictability for sinus floor augmentation and dental implant placement. The purpose of this retrospective study was to evaluate the outcomes of dental implant placement in sinus-augmented areas with preexisting sinus mucosal thickening.

**Methods:**

This study involved the review of cone-beam computed tomographic (CBCT) scans taken on patients that underwent both maxillary sinus elevation with grafting and implant placement at the University of Michigan School of Dentistry from 2004 to 2014. Cases with documented radiographic and clinical follow-up were included. The data analyses revealed the following.

**Results:**

A total of 29 CBCT scans met the inclusion criteria for evaluation, and 93.1% of them had maxillary sinus mucosal/tissue thickening. Specifically, 6.9% of cases exhibited no thickening, 6.9% had minimal thickening (1–2 mm), 20.7% of cases had moderate thickening (2–5 mm), and 65.5% had severe thickening (>5 mm). We propose these categorical measurements of tissue thickening as a new “mucosal thickening index.” The tissue thickening did not vary based on gender, age, or smoking status, nor did it relate to the underlying alveolar ridge height. However, patients with a history of periodontal diseases demonstrated a significant association with mucosal thickening (*p* = 0.0043). These data indicate that there is high implant and grafting success rate (100%) in the maxillary sinus despite large and varied physiologic sinus mucosal/tissue thickening.

**Conclusions:**

Based on study findings, this research will help guide dental practitioners regarding cases that exhibit mucosal thickening. These data support the concept that physiologic mucosal thickening in varied ranges is not associated with implant or grafting failure in the maxillary sinus.

## Background

Despite the high survival rate of dental implants inserted in maxillary sinuses that have undergone sinus floor elevation (SFE) with bone grafting, complications still occur [1–3]. Sinus membrane perforation is reported to be the most common complication [4, 5]. Postoperative maxillary sinusitis is less common (0–22%) [6, 7]; nevertheless, it could potentially compromise the outcome of SFE and affect the overall well-being of the patient [8]. Developing postoperative sinusitis is often associated with a reduction in the patency or complete obstruction of the ostium due to inflammatory edema in the sinus or preexisting chronic sinusitis [8–12].

Various cyst-like pathologies can be found in the maxillary sinus, including a pseudocyst and a surgical ciliated cyst [13]. However, a thickened mucous membrane can be physiologic or benign without presentation of symptoms. In contrast, cyst-like entities may need surgical removal due to their pathologic progression [14]. A surgical ciliated cyst is defined as a posterior maxillary cyst found after the surgical treatment of maxillary sinusitis [15]. Pseudocysts are diagnosed as dome-shaped, noncorticated soft tissue opacities with a well-defined border in the maxillary sinus [16].

In the dental literature, sinusitis has most commonly been identified on radiographs as thickening of the sinus membrane [12]. Mucosal thickening >2 mm is considered a threshold for pathological thickness [17]. Although mucosal thickening is the most common radiographic finding observed regarding sinus pathology [18], the knowledge regarding its clinical significance on the outcomes of dental implants and grafting in the maxillary sinuses is still limited [19].

The purpose of this retrospective study was to evaluate the outcomes of dental implant placement in sinus augmented areas with preexisting mucosal thickening of more than 2 mm. The aims of this study were to (1) determine the success rate of dental implant placement in augmented maxillary sinus areas with mucosal thickening, (2) evaluate the effect of gender, age, and smoking on the dimensions of sinus mucosal membranes, and (3) based on the overall findings, develop a written protocol to guide dental practitioners regarding cases that exhibit mucosal thickening (the threshold of safety).

## Methods

### Study design

Our study hypothesis was that mucosal thickening of more than 2 mm and up to 1/3 of the volume of the sinus would not alter the predictability for SFE and dental implant placement. The primary outcome was to determine the success rate of dental implant placement in augmented maxillary sinus areas with mucosal thickening. A secondary outcome was to evaluate the effect of gender, age, and smoking on the dimensions of sinus mucosal membranes.

This study consisted of performing a retrospective analysis of cone-beam computed tomographic (CBCT) scans taken with a CBCT machine (i-CAT Cone-Beam Computed Tomography machine, Imaging Sciences International, Hatfield, PA) for patients that underwent both maxillary sinus elevation with grafting and implant placement at the University of Michigan School of Dentistry from 2004 to 2014. This study was approved by the University of Michigan Institutional Review Board.

### Subject inclusion and exclusion criteria

Subjects that exhibited the following criteria were included in the study: partial edentulism, over 18 years old, received dental implants after sinus grafting, and had clinical and radiographic follow-up. These subjects had at least one CBCT scan prior to a SFE procedure. Subjects that exhibited the following criteria were excluded from the study: under 18 years old, subjects whose CBCT images were not clear enough to read, or had portions of the maxillary sinus not fully captured in the field of view. Subject data that was extracted from the general and medical record included the following: age, gender, and any systemic issues following a review of overall systems for the presence of any pathology (respiratory system, cardiovascular system, diabetes status, smoking history, etc.).

Subject data extracted from the dental records included the following: restorative, endodontic, periodontal, orthodontic, and oral surgery treatment or extractions.

Given these specific inclusion and exclusion criteria and the specific purpose of this study, only 29 cases qualified for inclusion from an original screen of approximately 4000 cases. An initial search of our database resulted in a larger number of cases that would theoretically qualify; however, further investigation revealed the need to exclude a great number of cases. The reasons for exclusion of these cases were as follows: scatter on the CBCT images due to fixed prosthodontics, unclear CBCT images, poor charting that did not allow for proper data gathering, no follow-up radiographs, not enough of the sinus being visible in the image, diagnosed periapical pathosis in the examined areas, implants not being placed in the area of the maxillary sinus, or no grafting completed in the maxillary sinus. Although these factors greatly reduced our sample size, this, in turn, created a stronger data set for analyses.

### Subject privacy protection

The study required access to University of Michigan Protected Health Information (PHI). PHI was necessary in order to track and coordinate the CBCT data and dental and medical history for each subject. Corresponding subject charts and electronic records were reviewed for retrieval of relevant implant placement and restorative history, medical history, and demographic information, including gender and age and smoking history. Also, any pertinent dental treatment was received and response to treatment were reviewed and recorded. No other personal information was retrieved. The use of PHI involved no greater than minimal risk because each subject was assigned a coded number that was used for all data analyses, tables, and reports.

### Measurement methodology

Using CBCT images, the mucosal thickness/height was measured at the point of maximum height using sagittal views, which were perpendicular to the underlying sinus floor at edentulous sites [12, 20]. Using these sagittal views, measurements were taken at four points: ¼, ½, and ¾ of the widest distance of the maxillary sinus from anterior to posterior. In order to standardize the measurements for each sinus, each scan was carefully oriented in the axial, coronal, and sagittal plane. In the axial plane, a horizontal line from the right and left zygoma was chosen as the standard. Orienting the hard palate horizontally was the standard in the coronal plane as well as in the sagittal plane. The specific teeth that were to be replaced by implants were then located by reviewing each patient’s chart, and the area of implant placement was located in the CBCT. To select the appropriate slice in the sagittal view to measure the mucosal thickening, the vertical line in the axial view was placed in the center of the alveolus where the future implant was to be placed. The appropriate sagittal view was then obtained and measurements of the mucosal thickening were performed. Each measurement was completed with the brightness and contrast set at 50%, and the zoom function was utilized to better visualize the soft tissue.

**Fig. 1:**
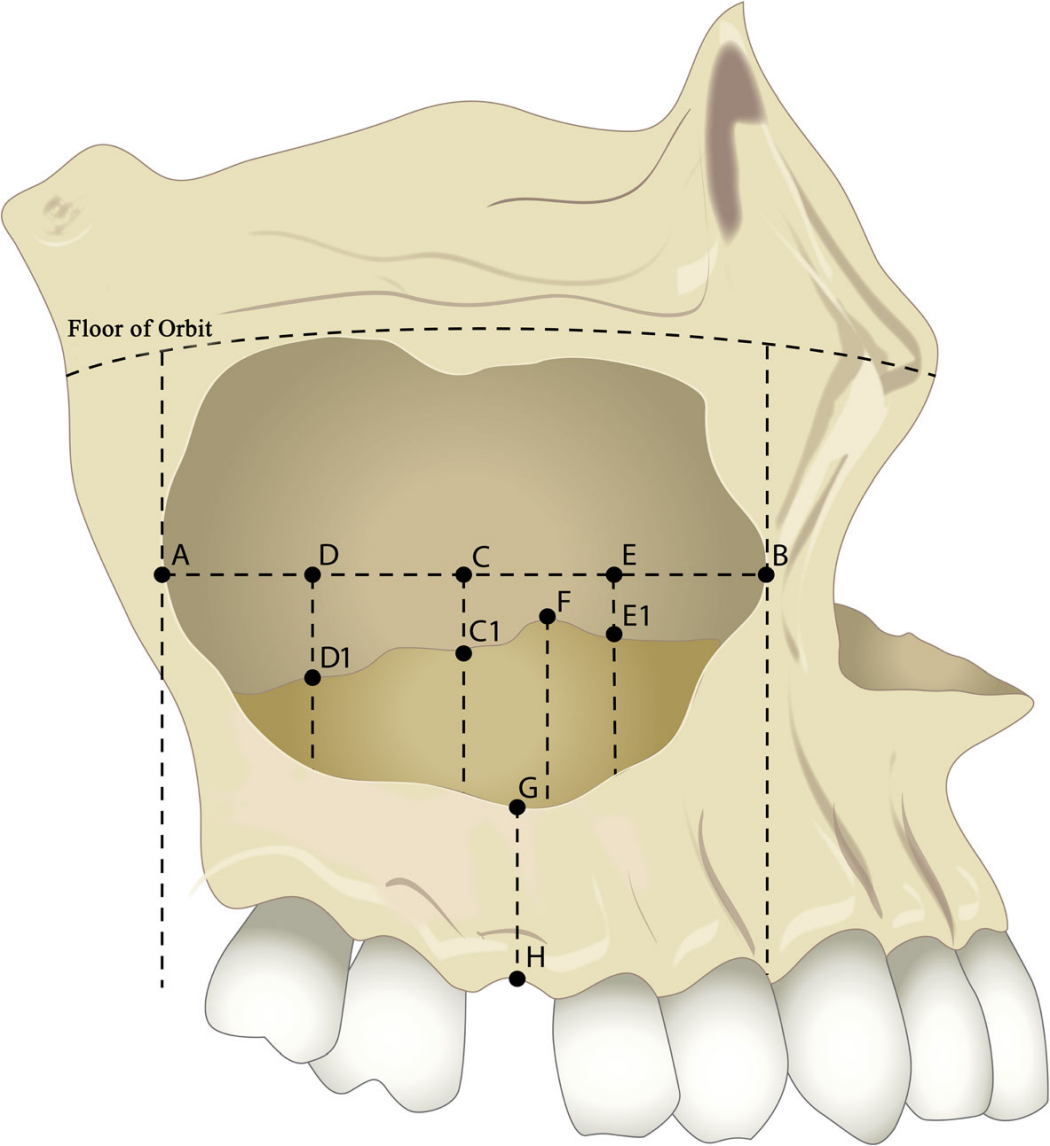
Figure illustrating the reference points of the CBCT measurements. *A*: most posterior point of the sinus wall; *B*: most anterior point of the sinus wall; *C*: mid-point between *A* and *B*; *C1*: measurement of mucosal thickening perpendicular to *A*–*B* line at point *C*; *D*: mid-point between *A* and *C*; *D1*: measurement of mucosal thickening perpendicular to *A*–*B* line at point *D*; *E*: mid-point between *B* and *C*; *E1*: measurement of mucosal thickening perpendicular to *A*–*B* line at point *E*; *F*: highest extension of thickened sinus membrane; *G*–*H*: height of residual alveolar bone (measured at the mid-point of the edentulous ridge/implant-planned site)

The sites that were measured are specified in the image below (Fig. 1). The most posterior and anterior aspects of the visible maxillary sinus were measured. The ½ point along with the ¼ and ¾ points were then selected, and the measurements of the mucosal thickening were then completed at these three sites. The thickest portion of the mucosa was also measured if it did not coincide with one of the three earlier measurements. Post implant placement radiographs were analyzed to locate the area where the implant was placed, and this area was then estimated on the CBCT and the thickness of the alveolus was measured there.

### Statistical analysis

Outcome analyses included the overall implant survival rate and percentage of mucosal thickening at different sites. The associations between the amount of mucosal thickening and the recorded variables, including patients’ systemic conditions and dental history, were estimated by linear mixed models. Adjustment for potential inter-variable influence using regression analysis was also performed. A *p* value of 0.05 was used as the level of significance. All the statistical analyses were calculated using a computer program (SAS Institute Inc. 2011. Base SAS® 9.3 Procedures Guide, Cary, NC).

## Results

Twenty-nine CBCT images (11 females and 18 males) were included in this study. All the implants placed in these included cases survived, representing a 100% implant survival rate. With regards to measurements of mucosal thickening, the intra-examiner reproducibility revealed an exact intra-examiner correlation of 99%, with a *p* value of 0.40 for a *t* test for independent samples, indicating a high reproducibility and intra-examiner agreement for the radiographic measurements. The study data for the 29 analyzed CBCT scans are presented in Table 1. The mean follow-up time after implant placement was 3.3 ± 2.2 (range 1 to 7) years.

**Table 1:** CBCT measurements of sinus mucosal thickening

Patient	Anterior (E1-floor of the sinus)	Middle (C1-floor of the sinus)	Posterior (D1-floor of the sinus)	Thickest (F-floor of the sinus)
1	3.06	0.32	0.76	4.59
2	0.34	0.21	0.20	0.34
3	0.39	0.54	1.38	1.66
4	4.15	3.79	0.61	6.36
5	5.64	1.33	3.73	8.42
6	7.34	0.77	0.86	7.66
7	1.93	9.25	6.17	12.5
8	8.22	7.89	1.62	12.27
9	3.52	10.05	4.76	10.05
10	7.62	0.43	2.03	8.11
11	7.19	2.75	0.77	7.62
12	0.26	9.26	8.38	9.26
13	0.76	19.50	0.46	22.81
14	14.95	11.33	9.67	18.92
15	0.79	0.71	1.36	1.55
16	0.60	3.34	1.52	4.40
17	4.44	1.82	3.60	12.25
18	1.39	0.68	0.52	8.00
19	0.21	0.24	0.24	0.24
20	16.27	11.11	8.07	16.27
21	2.87	5.10	6.40	7.85
22	5.13	0.64	4.12	5.13
23	1.19	1.36	0.25	2.48
24	0.80	2.39	2.08	4.84
25	19.05	16.94	10.14	20.10
26	3.51	4.25	2.63	4.25
27	0.28	1.62	2.85	2.85
28	9.94	7.21	5.29	11.07
29	2.56	6.28	9.98	9.98
Mean (mm)	4.63	4.87	3.46	8.34
Standard deviation (mm)	4.95	5.10	3.17	5.70

### Percentage and amount of mucosal thickening

Among the subjects, 93.1% of patients had maxillary sinus mucosal/tissue thickening. Specifically, 6.9% of cases exhibited no thickening (≤1 mm), 6.9% had minimal thickening (>1 mm but ≤2 mm), 20.7% of cases had moderate thickening (>2 mm but ≤5 mm), and 65.5% had severe thickening (>5 mm). However, only 45.9% of designated implant sites presented sinus mucosal thickening. The average amount of mucosal thickening in the anterior section (point E1 to floor of the sinus) was 4.63 ± 4.95 mm, in the middle section (point C1 to floor of the sinus) it was 4.87 ± 5.10 mm, and in the posterior section (point D1 to floor of the sinus) it was 3.46 ± 3.17 mm. The average mucosal thickening (point F to floor of the sinus) was 8.34 ± 5.70 mm, and it ranged from 1.55 to 22.81 mm.

### Factors associated with mucosal thickening

A significantly higher amount of mucosal thickening was associated with patients with a history of periodontal diseases (*p* = 0.004). Other factors, such as gender (*p* = 0.054), and systemic factors, including respiratory diseases (*p* = 0.313), cardiovascular diseases (*p* = 0.438), diabetes (*p* = 0.209), or smoking (*p* = 0.541), were not significantly associated with mucosal thickening.

In terms of dental history, the presence of tooth restorations (*p* = 0.056), endodontic treatment (*p* = 0.379), orthodontic treatment (*p* = 0.125), edentulism (*p* = 0.718), and underlying alveolar ridge height (point G to H, *p* = 0.889) were not associated with mucosal thickening. The results of the statistical analyses after inter-variable adjustment are presented in Table 2.

**Table 2:** Statistical results after inter-variable adjustment showing the association between recorded parameters and sinus mucosal thickening; *p* values that showed statistically significant differences are italicized

	Gender	Respiratory diseases	Cardio-vascular diseases	Diabetes mellitus	Smoking	History of periodontal diseases	Endodontic treatment	History of orthodontic treatment	Alveolar ridge height	Extraction performed
*p* value	0.054	0.3130	0.4376	0.2090	0.5413	*0.0043*	0.3793	0.1248	0.8896	0.7175

## Discussion

**Fig. 2:**
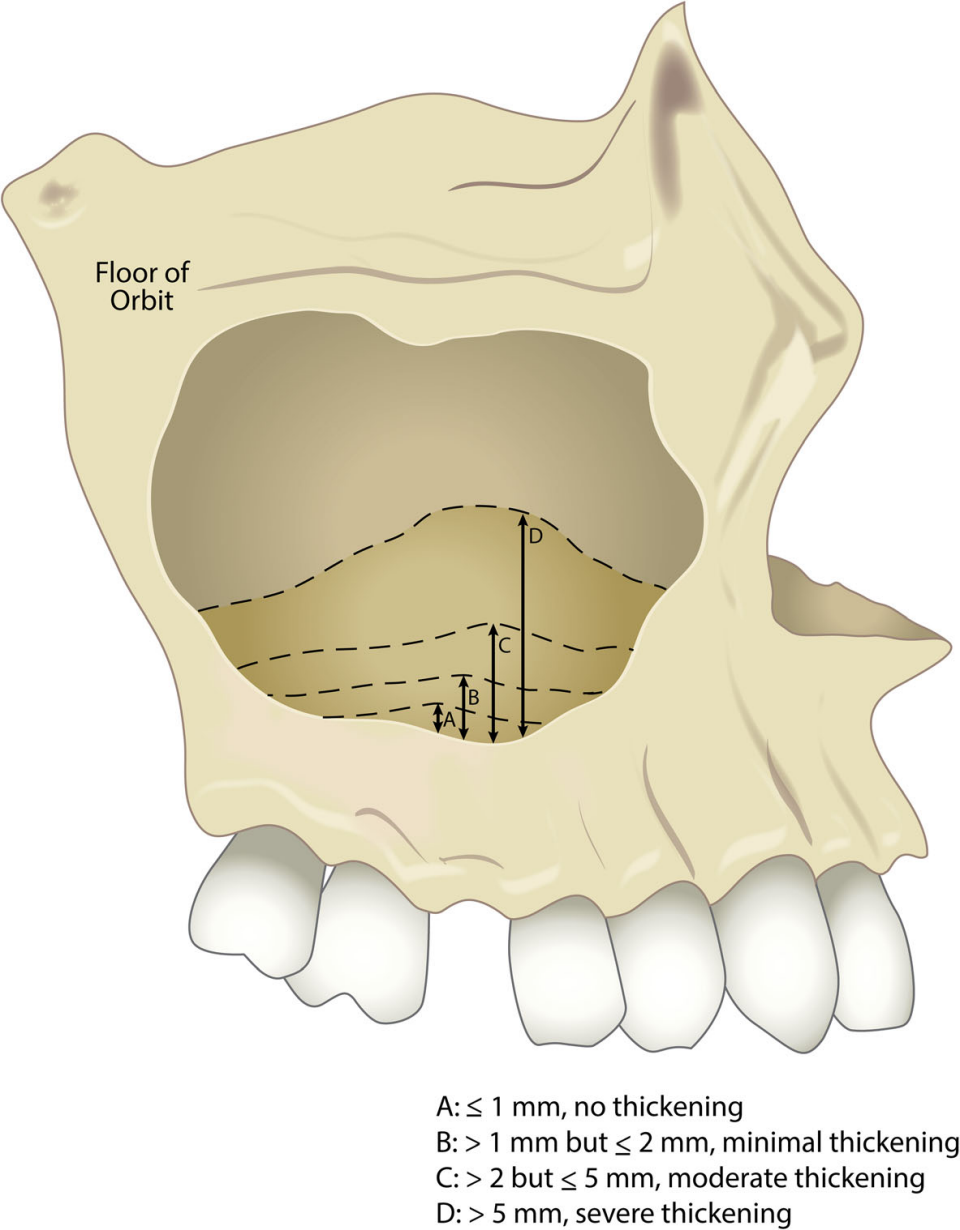
Figure illustrating the proposed mucosal thickening index. *A* ≤1 mm, indicating no thickening; *B* >1 mm but ≤2 mm, indicating minimal thickening; *C* >2 mm but ≤5 mm, indicating moderate thickening; *D* >5 mm, indicating severe thickening

CBCT imaging has been recognized as a more sensitive imaging modality for identifying sinus thickening and pathoses in the posterior maxilla compared to panoramic radiography [21, 22]. This could explain why the current study identified a higher prevalence of mucosal thickening compared to earlier studies [23]. However, compared to other similar CBCT studies [21, 24, 25], the prevalence reported in the current study is still much higher than the previously published articles with a similar study design. Ritter et al. [25] in a retrospective CBCT study reported 38.1% of subjects presented with mucosal thickening. Similarly, Pazera et al. [24] reported a prevalence of 23.7% and Janner et al. [20] reported 37% of cases with membrane thickening. In a recent study, Brüllmann et al. [17] found 74% of evaluated sinuses had sinus findings upon CBCT examination. These differences in prevalence might result from the different inclusion criteria among the studies. Another potential explanation for these differences in the reported prevalence of mucosal thickening may be due to the ambiguous definition used in the early studies. Some studies suggested that 2 mm should be considered the threshold for identifying mucosal thickening [20], and thus the prevalence of slight mucosal thickening may have been underestimated. To avoid this situation, the current study analyzed the thickness of sinus membranes based on four different categories: no thickening (≤1 mm), minimal (>1 mm but ≤2 mm), moderate (>2 mm but ≤5 mm), and severe thickening (>5 mm). According to this index (Fig. 2), 93.1% of cases examined had maxillary sinus mucosal/tissue thickening and 65.5% had severe thickening.

The current study demonstrated that sinus mucosal thickening does not correlate with implant survival. This result is consistent with a previously published report by Jungner et al. [26]. In their study, the presence of sinus thickening was not significantly associated with implant failure. Similarly, our study found a 100% implant survival rate for both patients with and without sinus mucosal thickening. It is worth mentioning that none of the CBCT images evaluated in the current study showed signs of sinusitis or periapical pathoses, despite indications of physiologic mucosal thickening. All the selected images presented clear sinuses without signs of infection. Therefore, it could be postulated that physiologic mucosal thickening does not contribute to implant failure. However, an association between pathologic mucosal thickening and implant survival cannot be drawn. If sinusitis is suspected, it is suggested that clinicians consult the appropriate medical specialists before implant placement.

Based on the findings of the current study, a history of periodontal disease is the only identified parameter significantly associated with sinus mucosal thickening. This finding indicates that clinicians should expect some degree of mucosal thickening when performing sinus augmentation procedures in a previously periodontally involved site. This finding is consistent with several previously published studies [27–29]. Phothikhun et al. [28] reported that sinuses with severe periodontal bone loss were three times more likely to have mucosal thickening. In a more recent study, Ren et al. [29] reported an odds ratio of 4.62 for patients with severe periodontal bone loss with mucosal thickening. A possible explanation for this phenomenon is that increased inflammatory cytokines resulting from periodontal diseases might also reach the maxillary sinus, and thereby trigger an increased membrane thickening. With regards to implant treatment outcomes, while our study found that there is no association between mucosal thickening and future implant survival, a higher chance of sinus membrane perforation during sinus lift procedures has been reported when a thicker membrane is present [3, 30].

Our study did not find a significant association between endodontically treated teeth and mucosal thickening. Though this finding is consistent with some previously published studies [27, 28], other studies [21, 31] did report an association. These discrepant findings could be the result of different inclusion criteria in the study design. Since our study did not include any patients with radiographic signs of pulpal pathoses, these data suggest that successful root canal treatment without signs of apical radiolucency should not be considered as a risk indicator of future mucosal thickening. On the other hand, it has been reported [32, 33] that the presence of apical periodontitis is related to sinus mucosal thickening, which should alert clinicians when planning future implant-related procedures. Because periapical infections are considered a multifactorial entity, they should be carefully evaluated and treated to ensure a favorable implant treatment outcome [34, 35]. In addition, the influence of a periapical scar of dense collagen tissue, formed after conventional root canal treatment, on implant treatment outcomes has not yet been fully explored. Therefore, additional future investigations are needed to examine these unresolved issues.

Although residual alveolar ridge height has been associated with sinus mucosal thickening [36], our study did not find a significant association between these two parameters. Acharya et al. [36] reported that lower available bone height in the subsinus region was related to thickened sinus membranes within an Asian-Indian and Hong Kong-based Chinese population. Differences in the ethnic composition and geographic location of this population might explain the different findings compared to our study, which was primarily comprised of a Caucasian cohort in North America. Also, in their study, the majority of patients (80.53%) had some degree of periodontal disease, which might have directly influenced their outcome analysis. Since residual bone height depends highly on the rate of bone remodeling and sinus pneumatization after tooth extraction [37], future prospective clinical trials are needed to investigate the relationship between changes in maxillary sinus dimension and mucosal thickness.

This study presents new data on maxillary sinus mucosal thickening derived from a carefully defined data set; however, there were some limitations in the study. One limitation was the limited sample size. However, as discussed, our stringent case selection criteria yielded a more uniform data set for analyses. Other limitations were related to the actual measurements of the maxillary sinus. In order to normalize the data, specific planes were used as the basis for measurements. However, due to anatomical variations in patients, it was not always possible to orient each plane in the exact position. In those instances, the plane of orientation was set as close to ideal as possible. For example, it was not possible to orient the entire hard palate horizontally in some patients if it had a curvature. In addition, since the maxillary sinus is a three-dimensional structure with many variations among patients, situations arose where mucosal thickening extended from the septa or lateral walls instead of just on the floor of the sinus. In these situations, best judgment was utilized in order to decide if these areas of thickening would have an impact on the implant placement area. Also, variations in the anatomical features made orienting CBCT scans at times challenging, and this may have influenced the study outcomes.

## Conclusions

Our study found that the largest tissue thickening was present in the middle section of the maxillary sinus. This tissue thickening did not vary based on gender, age, or smoking status, nor did it relate to the underlying alveolar ridge height. However, patients with a history of periodontal diseases demonstrated a significant association with mucosal thickening. A mucosal thickening index was proposed as a guide for future studies and clinical practice. A high implant and grafting success rate (100%) in the maxillary sinus was noted despite large and varied physiologic sinus mucosal/tissue thickening.

## Abbreviations

CBCT: Cone-beam computed tomographic
PHI: Protected Health Information
SFE: Sinus floor elevation
